# Comparison of desferrioxamine and NODAGA for the gallium-68 labeling of exendin-4

**DOI:** 10.1186/s41181-019-0060-9

**Published:** 2019-05-16

**Authors:** Simon A. M. Kaeppeli, Roger Schibli, Thomas L. Mindt, Martin Behe

**Affiliations:** 1 Center for Radiopharmaceutical Sciences ETH-PSI-USZ, Paul Scherrer Institute, OIPA/102, Forschungsstrasse 111, 5232 Villigen-PSI, Switzerland; 2 Department of Chemistry and Applied Biosciences, ETH Zurich, Vladimir-Prelog-Weg 4, 8093 Zurich, Switzerland; 3 Ludwig Boltzmann Institute Applied Diagnostics, General Hospital Vienna (AKH), c/o Sekretariat Nuklearmedizin Währinger Gürtel 18-20, Vienna, Austria; 4 Department of Biomedical Imaging and Image Guided Therapy, Division of Nuclear Medicine, Medical University of Vienna, Vienna, Austria

**Keywords:** Exendin-4, β-cells, Insulinoma, GLP-1R, NODAGA, DFO

## Abstract

**Introduction:**

Radiolabeled exendin-4 (Ex4) derivatives are used to target the glucagon-like peptide-1 receptor (GLP-1R) for the clinical diagnosis of insulinomas, a rare type of neuroendocrine tumor. Gallium-68 is an ideal diagnostic nuclide for this application and a study evaluating an exendin-4-NODAGA conjugate is currently underway. However, in complexion with the chelator DFO, its in vivo stability has been a matter of dispute. The aim of this work was to directly compare [^68^Ga]Ga-Ex4NOD with [^68^Ga]Ga-Ex4DFO in vitro and in vivo.

**Methods:**

In our approach, we directly compared *N′*-[5-(acetyl-hydroxy-amino)pentyl]-*N*-[5-[3-(5-aminopentyl-hydroxy-carbamoyl)propanoylamino]pentyl]-*N*-hydroxy-butane diamide (desferriox-amine B, DFO) and 2-(4,7-bis (carboxymethyl)-1,4,7-triazonan-1-yl) pentanedioic acid (NODAGA) conjugated to exendin-4 in vitro and in vivo*.* We radiolabeled the peptides with gallium-68, followed by HPLC quality control. In vitro characterization was performed in CHL cells overexpressing the GLP-1R and in vivo studies were conducted with CD1 nu/nu mice carrying tumors derived from these cells.

**Results:**

We found that both peptides could be radiolabeled with a molar activity of about 9.33 MBq/nmol without further purification. They internalized equally well into GLP-1R-expressing cells and their IC_50_ was similar with 15.6 ± 7.8 nM and 18.4 ± 3.0 nM for [^nat^Ga]Ga-Ex4NOD and [^nat^Ga]Ga-Ex4DFO, respectively. In vivo, [^68^Ga]Ga-Ex4NOD accumulated more in all tissue, while [^68^Ga]Ga-Ex4DFO exhibited a more favorable target-to-kidney ratio.

**Conclusion and relevance:**

DFO is a suitable chelator for the radiolabeling of exendin-4 derivatives with gallium-68 for in vitro and preclinical in vivo studies. DFO performed better in vivo due to its significantly lower kidney accumulation (*p* < 0.0001). It was also found to be stable in vivo in mice, contrary to earlier reports. Based on our results, the DFO chelating system in combination with exendin-4 would be an interesting option for clinical imaging of insulinomas.

**Electronic supplementary material:**

The online version of this article (10.1186/s41181-019-0060-9) contains supplementary material, which is available to authorized users.

## Background

Insulinomas, a form of neuroendocrine tumors originating from pancreatic β-cells and are usually benign. However, in a minority of cases malignant tumors are found at time of diagnosis (Hirshberg et al. 2000; Service et al. 1991). Insulinomas belong to the functional type of pancreatic neuroendocrine tumors (PNET) as they secrete insulin (Burns and Edil 2012). Due to this autonomous insulin secretion, hypoglycemia is a major concern in patients and the complete surgical removal of such tumors is the favored treatment (Burns and Edil 2012).

Insulinomas generally express highly and specifically the glucagon-like peptide-1 receptor (GLP-1R) as they are of β-cell origin (Reubi and Waser 2003). GLP-1R is a G-protein coupled receptor of the class B receptor family (Zhang et al. 2017). Its endogenous peptide ligand, GLP-1, has a very short biological half-life of about 2 min. Dipeptidyl peptidase-4 (DPP4) is responsible for the cleavage of the peptide (Baggio and Drucker 2007). GLP-1R can also be targeted by several more stable GLP-1 derivatives, such as liraglutide and semaglutide, and analogues, such as lixisenatide and exendin-4. All of them are clinically used in non-radiolabeled form for the management of type 2 diabetes (Aroda 2018).

Currently, exendin-4 coupled to the chelator 2-(4,7-bis (carboxymethyl)-1,4,7-triazonan-1-yl) pentanedioic acid (NODAGA) is in clinical evaluation of adult hyperinsulinemic hypoglycemia (EudraCT Number: 2014–003167-38) (^68^Ga-NODAGA-exendin-4 PET/CT for Diagnostic Imaging in AHH n.d.). Furthermore, NODAGA is used in preclinical imaging studies with gallium-68 and it is also frequently investigated with copper-64, another interesting PET radionuclide with a half-life of 12 h, which has been used previously with exendins and other peptidic tracers (Nedrow et al. 2014; Mikkola et al. 2014). *N′*-[5-(acetyl-hydroxy-amino)pentyl]-*N*-[5-[3-(5-aminopentyl-hydroxy-carbamoyl)-propanoylamino]pentyl]-*N*-hydroxy-butane diamide (desferrioxamine B, DFO) DFO has mainly been investigated as a chelator for zirconium-89 labeling of antibodies (immunoPET) (Verel et al. 2003; Mayer et al. 2017; Brandt et al. 2018).

We previously described an exendin-4 tracer conjugated to the chelator DFO for use with either the short-lived gallium-68 or the longer-lived PET nuclide zirconium-89, for the imaging at later timepoints or in combination with radioguided surgery (Bauman et al. 2015). We could show that the tumor uptake with [^68^Ga]Ga-Ex4DFO was higher than with the clinically tested SPECT tracer [^111^In]In-DTPA-exendin-4.

In the present study, we expanded on previous encouraging findings made with DFO-exendin-4 and investigated how NODAGA-exendin-4 compared in vitro and in vivo when used with the standard PET nuclide gallium-68. A gallium-68 labeled NODAGA-conjugated exendin-4 derivative is currently investigated in clinics for the diagnosis of adult hyperinsulinemic hypoglycemia and insulinoma (^68^Ga-NODAGA-exendin-4 PET/CT for Diagnostic Imaging in AHH n.d.). A prevailing issue with radiometal labeled exendin-4 derivatives is the persistent kidney accumulation (Simonsen et al. 2006; Eriksson et al. 2016). Insulinomas are frequently located within the pancreas and therefore in close proximity to the kidneys. SPECT imaging has been shown not to achieve sufficient resolution to detect them. Instead, PET is the favored imaging modality with its higher resolution, as shown very clearly by Antwi and colleagues (Antwi et al. 2015). Therefore, we also aimed to evaluate Ex4DFO and Ex4NOD with respect to their accumulation in the kidneys as chelators are known to have a substantial effect on the biodistribution of a tracer (Price and Orvig 2014).

## Methods

### Peptides and labeling

Exendin-4 was conjugated to *N′*-[5-(acetyl-hydroxy-amino)pentyl]-*N*-[5-[3-(5-aminopentyl-hydroxy-carbamoyl)propanoylamino]pentyl]-*N*-hydroxy-butane diamide (desferrioxamine, DFO) to give rise to [Lys^40^-(Ahx-DFO)NH_2_]exendin-4 as described in (Bauman et al. 2015), in short Ex4DFO. In the second derivative, exendin-4 was modified at position 14 where methionine (Met) was replaced with Norleucine (Nle) to improve oxidative stability during labeling (Velikyan et al. 2017). Then, it was conjugated to 2-(4,7-bis (carboxymethyl)-1,4,7-triazonan-1-yl) pentanedioic acid (NODAGA) to give rise to [Nle^14^-Lys^40^-(NODAGA)NH_2_]exendin-4, in short Ex4NOD. It was synthesized commercially (piChem, Graz, Austria). The schematic structures of the peptides are shown in Additional file 1: Figure S1.

For the radiolabeling, gallium-68 was freshly eluted with 0.1 M HCl (Rotem Industries, Israel) from a IGG100 ^68^Ge/^68^Ga Generator (Eckert & Ziegler, Berlin, Germany) through a plastic needle from a generator into a glass vial, which was prewashed with metal-free 0.1 mM HCl and flushed with metal free analytical grade water (Sigma-Aldrich, Buchs, Switzerland). Typically, an activity of about 950 to 990 MBq in 6.5 to 7.0 mL was obtained (activity concentration of about 140 MBq/mL). Briefly, the labeling was performed as follows. Six μL of peptide (250 μM) was added to 50 μL of 1 M ammonium acetate (pH 5.5) and 2 μL of 3 M NaOH in a 1.5 mL screw-top tube. Then, 100 μL of gallium-68 (14 MBq) was added and the mixture was incubated at 95 °C for 10 min in a heating block, after which it was spun down. One μL of the labeling mixture was diluted into 150 μL of 1% ethylenediaminetetraacetic acid (*EDTA*) in water and analyzed by reversed-phase high performance liquid chromatography (RP-HPLC) on a ReproSil-Pur C18-AQ column (100 × 4.6 mm, 3 μm, Dr. Maisch, Germany). The column was eluted with H_2_O containing 0.1% trifluoroacetic acid (TFA), with a linear gradient from 15%–55% of acetonitrile containing 0.1% TFA for 10 min, followed by a linear gradient from 55%–70% for 5 min. The peptide peaks were observed at about 9.40 min (Ex4NOD) and 9.20 min (Ex4DFO) and the yields and purity of labeled peptides were typically ≥95%. No further purification was performed.

The labeling with the stable isotope was performed as described previously (Jodal et al. 2015). Briefly, 40 μL of the respective 250 μM peptide solution were added to 2 μL of a 10 mM [^nat^Ga]GaCl_3_ (Sigma-Aldrich, Buchs, Switzerland) solution and mixed with 60 μL of 0.5 M ammonium acetate buffer (pH 5.5). The reaction mixture was incubated at 75 °C for 30 min. 10 μL of 0.1 mM diethylenetriaminepentaacetic acid (DTPA) was added after incubation. The success of the labeling was verified by liquid chromatography-mass spectrometry (LC/MS) on an Atlantis C18 (250 × 4.6 mm; 5 μm) column on a Waters LCT Premier mass spectrometer (Milford, USA).

For the IC_50_ experiments, Ex4NOD was labeled with indium-111 as follows. Three MBq of [^111^In]InCl_3_ was added to the reaction mixture containing a final concentration of 10 μM of the peptide, 5 μL of 0.5 M ammonium acetate (pH 5.5) and 0.02 M HCl (adjusting the amount of [^111^In]InCl_3_ to 25 μL). The quality control was performed as described above. [^111^In]InCl_3_ was purchased from Mallinckrodt (Cham, Switzerland).

### Cell culture

Throughout the study Chinese hamster lung (CHL) cells stably transfected with human GLP-1R were used (a kind gift of Prof. Brigitte Lankat-Buttgereit). They were cultured in Dulbecco’s modified Eagle medium (DMEM) containing 4.5 g/L D-glucose, to which 10% fetal calf serum (FCS), 100 IU/mL penicillin G, 100 μg/mL streptomycin, 1.25 μg/mL fungizone®, 500 μg/mL geneticin sulfate, 1 mM sodium pyruvate, 0.1 mM non-essential amino acids and 2 mM L-glutamine were added. The cells were maintained at 37 °C in a humidified 5% CO_2_ atmosphere (Jodal et al. 2014, 2015). They were harvested with trypsin/EDTA. The trypsin was removed by centrifugation at 1000 g for 5 min.

### Competition binding (IC_50_) assay

To determine the half-maximal inhibitory concentration (IC_50_) of the exendin-4 derivatives, the cells were seeded in 12-well plates at 0.2 × 10^6^ cells per well and grown over night to reach a confluency of about 90–95%. The peptide under investigation was labeled with the stable isotope as described above, the reference peptide Ex4NOD was labeled with [^111^In]InCl_3_ as described above. The cells were washed twice with cold PBS (0.1 M NaCl, 0.042 M Na_2_HPO_4_, 0.011 M NaH_2_PO_4_) and incubated for 1 h on ice with the stably labeled peptide concentrations from 10^− 6^ to 10^− 11^ M and 150 to 200 pM of radiolabeled Ex4NOD. After binding, the cells were washed twice with PBS and solubilized with 1 M NaOH. The activity was measured using a γ-counter (Packard Cobra II Auto Gamma, PerkinElmer, Switzerland). The IC_50_ values were determined by fitting the data with non-linear regression using the least-squares fit of GraphPad Prism (version 7.0, GraphPad Software, La Jolla, USA). Significance of the calculated IC_50_ values was assessed in Prism using multiple t-test with the Holm-Sidak method. All experiments were performed in triplicates.

### Cell uptake assay

The cells were cultured as described for the IC_50_ assay and seeded into 6-well plates at a density of 0.75 × 10^6^ cells per well. They were probed with 150 to 200 pM labeled peptide and incubated at 37 °C for different timepoints (5, 15, 30, 60 and 120 min) at which the supernatant was collected. The cells were washed twice with 600 μL of PBS and the wash was pooled with the supernatant. The surface-bound peptide was collected from the cells after 5 min incubation with glycine buffer (0.1 M NaCl, 0.05 M Glycine, pH 2.8) at room temperature, during which the peptide detached from the receptors on the cell membranes. This fraction was collected separately and the glycine treatment was repeated. Finally, the cells were lysed by the addition of 1 M NaOH. In the latter samples, the internalized fraction of the radiolabeled peptide could be found. The non-specific binding and internalization were determined by blocking the receptors with 1 μM of commercially available, non-labeled exendin-4 (Bachem, Bubendorf, Switzerland), which was added to the control cells together with the radiolabeled peptide. The activity of the samples was measured in the γ-counter. The measurements were decay corrected and all experiments were performed in triplicates. Significance at the 2-h timepoint was analyzed with a one-way ANOVA and multiple comparisons of the three tracers were performed using Tukey’s multiple comparisons test with α = 0.05.

### Biodistribution

All animal experiments were conducted with permission of the local veterinary office and in accordance to the Swiss law of animal protection.

Six-week old female CD1 nu/nu mice were inoculated bilaterally in the shoulders with 8 × 10^6^ GLP-1R-expressing CHL cells (suspended in PBS, pH 7.4). Tumor growth was observed for 3 weeks after which the tumors weighed generally around 250 mg. The mice were randomly divided into the different study groups, four mice per group. All mice were injected with 100 kBq (8 to 10 pmol) of gallium-68-labeled peptide in 100 μl PBS via the tail vein. One group per peptide was co-injected with an excess of exendin-4 peptide (100 μg/mouse) to block specific tracer uptake. The mice were euthanized 0.5 or 1 h after injection of the tracer by CO_2_ inhalation. Organs (blood, heart, lungs, spleen, kidneys, pancreas, stomach, intestine, liver, muscle, bone) and tumors were collected in pre-weighted tubes. The activity was measured in the γ-counter. The values were decay corrected for evaluation. The percentage of injected activity per gram tissue (% i.A./g) was calculated for all collected tissues and analyzed in Excel (Microsoft, Redmond, USA). A two-way ANOVA was performed in GraphPad Prism, and comparisons of the two tracers was done using Tukey’s multiple comparisons test with α = 0.05. The target-to-kidney ratios were calculated in Microsoft Excel, and their averages, standard deviations, and significance were analyzed in Prism using two-way ANOVA as described above.

## Results

### Labeling of the peptides

Both, Ex4NOD and Ex4DFO could be radiolabeled with gallium-68 according to the protocol described above. The radiochemical yield was typically over 95% and a molar activity of 9.33 MBq/nmol for both peptides. Representative HPLC chromatographs of the radiolabeled peptides are shown in Additional file 1: Figure S2.

The labeling with stable gallium was achieved and representative MS analyses of the [^nat^Ga]Ga-labeled peptides are shown in Additional file 1: Figures S3 and S4.

There was no difference in the labeling outcome between Ex4NOD and Ex4DFO even though the latter still carries the potentially oxidizable methionine at position 14, which was replaced by neurleucine in Ex4NOD.

### Binding affinity to GLP-1R and receptor-mediated cell uptake

**Fig. 1:**
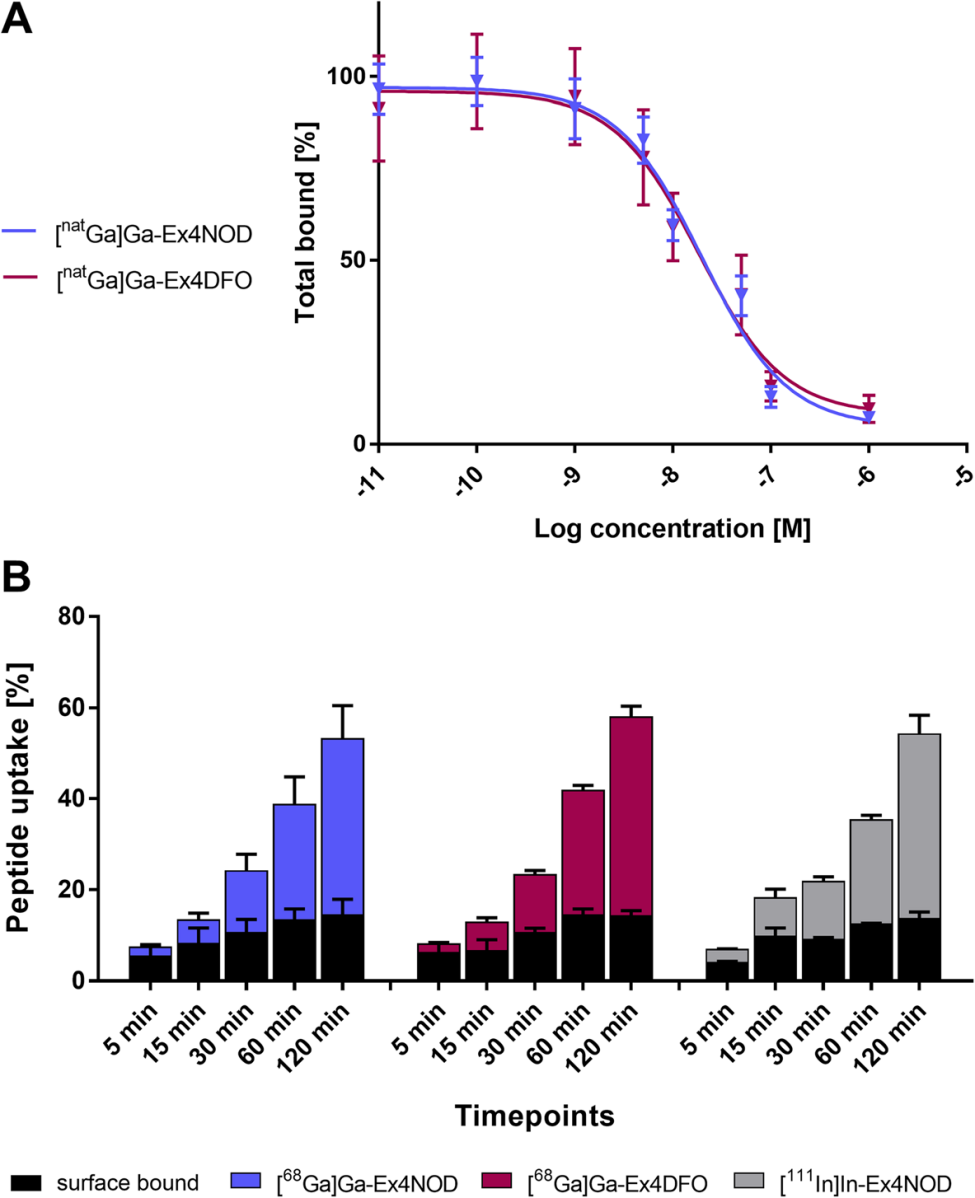
In vitro characterization of Ex4NOD and Ex4DFO (**a**) The IC_50_ curves of [^nat^Ga]Ga-Ex4NOD and [^nat^Ga]Ga-Ex4DFO, shown as the displacement of [^111^In]In-Ex4NOD from the GLP-1 receptor on CHL cells, correspond closely. **b** The GLP-1R-mediated cell binding and internalization show similar time-dependent trend in both gallium-68 labeled peptides, which again in turn is comparable to indium-111 labeled peptide. The percentage represents total cell uptake

We tested the affinity to the human GLP-1 receptor expressed in CHL cells in an IC_50_ competition assay using indium-111 labeled Ex4NOD peptide as reference and Ex4NOD and Ex4DFO labeled with stable gallium isotope in increasing concentrations. The IC_50_ of [^nat^In]In-Ex4NOD was determined to be 22.4 ± 2.9 nM. We found that [^nat^Ga]Ga-Ex4DFO had a slightly lower IC_50_ than [^nat^Ga]Ga-Ex4NOD, with 18.4 ± 3.0 nM and 19.6 ± 3.4 nM, respectively. However, the difference was statistically not significant. In fact, the IC_50_ curves of both peptides largely overlapped, as shown in in Fig. 1a.

Further, we investigated the pattern of GLP-1R mediated cell uptake in CHL cells. We observed an increase in internalization over 2 h, with a near-constant surface-binding, in particular towards later timepoints, as apparent in Fig. 1b. At 2 h, [^68^Ga]Ga-Ex4NOD reached 38.6 ± 7.4% internalization, whereas [^68^Ga]Ga-Ex4DFO was taken up to 43.6 ± 2.4% and the comparison peptide [^111^In]In-Ex4NOD showed 40.5 ± 4.2% internalization. The surface-binding was similar in all cases (14.5 ± 3.4%, 14.3 ± 1.2%, and 13.7 ± 1.5%, respectively).

### Timecourse biodistribution in GLP-1R tumor bearing mice

**Fig. 2:**
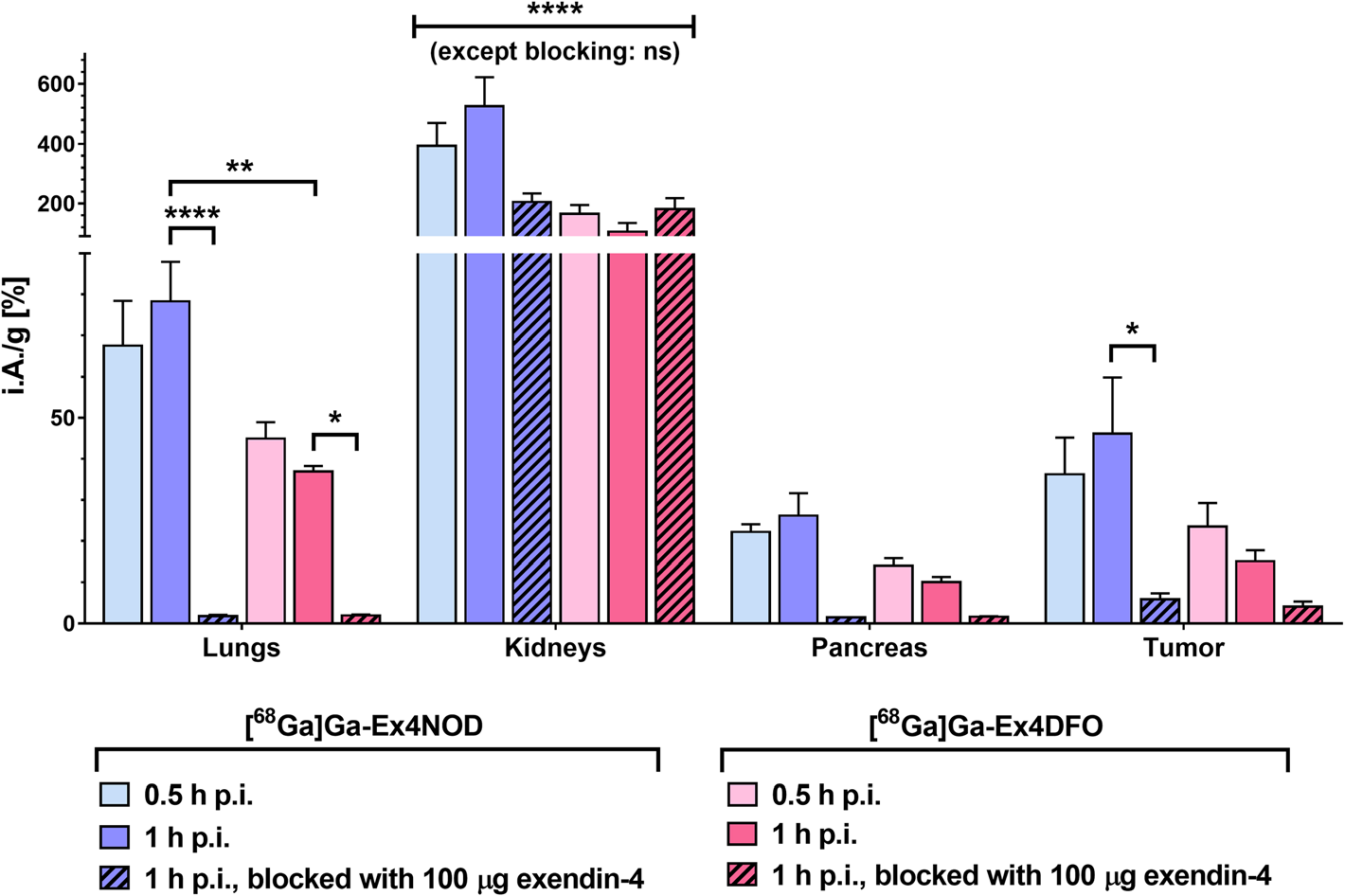
Selected organs from a timecourse biodistribution with [^68^Ga]Ga-Ex4NOD and [^68^Ga]Ga-Ex4DFO, performed in GLP-1R positive tumor-bearing CD 1 nu/nu mice (*n* = 4 per group). For both tracers, blocking at 1 h p.i. was achieved in all GLP-1R-expressing tissue

In the timecourse biodistribution study performed with both [^68^Ga]Ga-Ex4NOD and [^68^Ga]Ga-Ex4DFO, we found that [^68^Ga]Ga-Ex4DFO accumulated generally less all organs and tissues, including the GLP-1R-positive lung, pancreas, and tumor, as presented in Table 1. However, when comparing the tissues expressing GLP-1R such as the CHL tumors and the pancreas, no significant difference between the tracers could be found, except in the lungs, where the uptake at 1 h p.i. was significantly higher in [^68^Ga]Ga-Ex4NOD (Fig. 2). The kidney uptake in both timepoints was significantly higher in [^68^Ga]Ga-Ex4NOD, except for the blocking group, where no significant difference to [^68^Ga]Ga-Ex4DFO could be detected. Tumor uptake at 1 h p.i. appeared different, but did not reach significance (*p* = 0.0938).

**Table 1:** Full timecourse biodistribution study of gallium-68 labeled Ex4NOD and Ex4DFO, given as percent injected activity per gram tissue (% i.A/g)

	Ex4NOD	Ex4DFO	Ex4NOD	Ex4DFO	Ex4NOD	Ex4DFO
	0.5 h p.i.		1 h p.i.		1 h p.i., blocked	
Blood	1.98 ± 0.17	1.51 ± 0.12	0.86 ± 0.22	0.94 ± 0.11	0.86 ± 0.13	1.30 ± 0.19
Heart	2.00 ± 0.20	1.47 ± 0.10	1.53 ± 0.12	1.19 ± 0.14	1.73 ± 0.41	1.55 ± 0.25
Lung	67.61 ± 10.82	44.98 ± 4.01	78.30 ± 9.65	36.94 ± 1.34**	1.80 ± 0.32	1.94 ± 0.25
Spleen	1.79 ± 0.33	1.18 ± 0.21	1.58 ± 0.40	1.51 ± 0.08	2.11 ± 0.44	2.21 ± 0.38
Kidneys	393.24 ± 77.01	165.93 ± 29.22****	524.73 ± 97.35	105.27 ± 29.56****	204.74 ± 28.85	180.89 ± 37.02
Pancreas	22.21 ± 1.87	14.08 ± 1.75	26.17 ± 5.48	10.05 ± 1.25	1.46 ± 0.03	1.67 ± 0.08
Stomach	5.93 ± 2.48	3.27 ± 1.02	6.63 ± 1.78	2.05 ± 0.26	1.39 ± 0.18	1.66 ± 0.13
Intestines	3.71 ± 0.27	3.43 ± 0.49	4.09 ± 0.90	3.47 ± 0.88	1.56 ± 0.68	2.97 ± 0.54
Liver	1.50 ± 0.15	1.38 ± 0.14	1.56 ± 0.36	1.10 ± 0.25	0.98 ± 0.12	1.84 ± 0.10
Muscle	1.13 ± 0.07	0.82 ± 0.08	1.24 ± 0.35	0.96 ± 0.10	2.15 ± 0.52	1.88 ± 0.37
Bone	2.00 ± 0.17	1.39 ± 0.35	2.08 ± 0.87	1.79 ± 0.58	12.42 ± 11.02	9.51 ± 3.75
Tumor	36.29 ± 8.93	23.54 ± 5.70	46.18 ± 13.72	15.1 ± 2.67	5.89 ± 1.43	4.13 ± 1.22

The values are averages of four mice, given ± SD (α = 0.05), where ** is *p* < 0.01, and **** is *p* < 0.0001

We then proceeded to calculate the target-to-kidney ratio for the tumor, lungs, and pancreas and discovered that [^68^Ga]Ga-Ex4DFO significantly outperformed [^68^Ga]Ga-Ex4NOD in that respect, as evident from Table 2.

**Table 2:** GLP-1R-positive tissue-to-kidney and tumor-to-GLP-1R-positive tissue ratios derived from above timecourse biodistribution

	Ex4NOD	Ex4DFO	Ex4NOD	Ex4DFO
	0.5 h p.i.		1 h p.i.	
Lung-to-kidney	0.177 ± 0.041	0.275 ± 0.033	0.151 ± 0.013	0.371 ± 0.098
Pancreas-to-kidney	0.057 ± 0.007	0.087 ± 0.021	0.052 ± 0.019	0.099 ± 0.015
Tumor-to-kidney	0.092 ± 0.016	0.141 ± 0.017	0.089 ± 0.029	0.150 ± 0.044
Tumor-to-lung	0.546 ± 0.165	0.519 ± 0.083	0.594 ± 0.183	0.410 ± 0.078
Tumor-to-pancreas	1.627 ± 0.364	1.681 ± 0.391	1.787 ± 0.558	1.513 ± 0.310

## Discussion

Methods for high-resolution diagnostic imaging of insulinomas continue to be investigated pre-clinically and clinically. Exendin-4-based tracers, such as [^68^Ga]Ga-DOTA-Exendin-4 (Antwi et al. 2015), have already shown potential in clinical trials to replace less specific diagnostic tools, e.g. the [^111^In]In-DTPA-octreotide scintigraphy for the visualization of somatostatin receptor-positive tissue (Shi et al. 1998), or more general imaging modalities such as CT or MRI (Noone et al. 2005).

Based on our previous study investigating the chelator DFO for applications with the radiometal zirconium-89 for PET imaging (Bauman et al. 2015), we were curious how DFO would compare to NODAGA, which is, next to the clinically employed DOTA and the pre-clinically used DTPA (Antwi et al. 2015; Christ et al. 2015; van der Kroon et al. 2016), another chelator frequently used to pre-clinically investigate exendin-based tracers (Mikkola et al. 2014; Jodal et al. 2014).

There are conflicting reports about the stability of the gallium-chelation by DFO (Wester et al. 1997; Smith-Jones et al. 1994) and the complex has been observed to decompose during labeling at high temperatures (Tsionou et al. 2017). NODAGA on the other hand was demonstrated to label well with gallium-68 at elevated temperatures providing in vivo stable complexes (Asti et al. 2014). However, their thermodynamic complex formation stability constant (log *K*) have been reported to be very similar, with 28.65 for DFO and 29.63 for NOTA (Šimeček et al. 2011; Evers et al. 1989).

We found [^68^Ga]Ga-Ex4DFO to be stable, both during labeling, and during in vivo studies, as apparent from the liver uptake, which is comparable for both peptides. Additionally, we did not observe any oxidation of methionine in the quality control for the gallium-68 and the stable isotope labeling.

We established radiolabeling protocols that could be applied to both peptides, Ex4NOD and Ex4DFO, with equally good yields. We labeled the peptides at molar activities of about 9.3 MBq/nmol, which is in the range of what was previously used clinically (Antwi et al. 2015; Eriksson et al. 2014).

We then tested the tracers side-by-side for key features in vitro and in vivo and obtained nearly identical in vitro results, both for receptor affinity and cellular uptake, suggesting that the choice of chelator may not have a large impact in cell assays. However, these results need to be interpreted in the context of the cell model used, as previously, when cell uptake was tested in the rat insulinoma cell line RIN-m5F, [Lys^40^-(Ahx-DFO-^68^Ga)NH_2_]exendin-4 outperformed the reference compound [Lys^40^-(Ahx-DTPA-^111^In)NH_2_]exendin-4 (Bauman et al. 2015).

In our previous study, Rin-m5F did not strongly internalize exendin-4 based radiotracers in vitro, reaching less than 6% internalization after 2 h (Bauman et al. 2015). This is in contrast to CHL cells stably expressing the GLP-1 receptor where we reported internalization of around 40% with both gallium-67 and indium-111 (Jodal et al. 2014, 2015). In vivo, however, the results obtained with either cell line compare well with each other.

In vivo, we observed an overall higher kidney uptake with [^68^Ga]Ga-Ex4NOD, whereas the uptake of [^68^Ga]Ga-Ex4DFO was significantly lower and therefore resulted in a more favorable GLP-1R-positive tissue-to-kidney ratio. The high kidney uptake remains to be of concern and a wide variety of strategies are currently being developed to address this problem, such as by using fluorination and iodination approaches to circumvent the issue of residualizing radiometals (Mikkola et al. 2016; Lappchen et al. 2017). However, once a strategy is found to sufficiently decrease kidney uptake, the availability of a suitable, a versatile chelator for the use of radiometals will become central to the field of application of exendin-4 based tracers. Patients suffering from insulinomas, but also other conditions, such as hyperplasias of the pancreatic β-cell, could profit from such developments.

## Conclusion

In summary, we compared two gallium-68 labelled exendin-4 derivatives with DFO and NODAGA as a chelator. In vitro, both derivatives have similar cell uptake and affinity to GLP-1R. Nevertheless, overall, the DFO-functionalized peptide may be the better choice for imaging applications due to its significantly lower kidney uptake resulting in an improved target-to-kidney ratio.

To conclude, we believe this study should serve as a reference for consideration in future developments of exendin-4-based tracers for the targeting of GLP-1R, which will clearly remain of outmost importance.

## Additional file

Additional file 1: **Figure S1.** Schematic representation of the peptides investigated in the study. **Figure S2.** RP-HPLC chromatograph of [^68^Ga]Ga-Ex4NOD (top) and [^68^Ga]Ga-Ex4DFO (bottom). **Figure S3.** Mass spectrometric analysis of [^nat^Ga]Ga-Ex4NOD. **Figure S4.** Mass spectrometric analysis of [^nat^Ga]Ga-Ex4DFO. (DOCX 812 kb)

## Abbreviations

BSA: Bovine serum albumin
CHL: Chinese hamster lung
DFO: *N′*-[5-(acetyl-hydroxy-amino)pentyl]-*N*-[5-[3-(5-aminopentyl-hydroxy-carbamoyl)propanoylamino]pentyl]-*N*-hydroxy-butane diamide
DPP4: Dipeptidyl peptidase-4
DTPA: Diethylene triamine pentaacetic acid
EDTA: Ethylenediaminetetraacetic acid
Ex4: Exendin-4
Ex4DFO: [Lys^40^-(Ahx-DFO)NH_2_]exendin-4
Ex4NOD: [Nle^14^-Lys^40^-(NODAGA)NH_2_]exendin-4
FCS: Fetal calf serum
GLP-1R: Glucagon-like peptide 1 receptor
IC_50_: Half-maximal inhibitory concentration
LC/MS: Liquid chromatography-mass spectrometry
Met: Methionine
Nle: Norleucine
NODAGA: 2-(4,7-bis (carboxymethyl)-1,4,7-triazonan-1-yl) pentanedioic acid
PBS: Phosphate-buffered saline
PET: Positron emission tomography
PNET: Pancreatic neuroendocrine tumor
RP-HPLC: Reversed-phase high performance liquid chromatography
SPECT: Single-photon emission computed tomography
TFA: Trifluoroacetic acid
