# Supplementary material for: Comparison of desferrioxamine and NODAGA for the gallium-68 labeling of exendin-4
Source: EJNMMI Radiopharm Chem. 2019 May 16;4:9. doi: 10.1186/s41181-019-0060-9 (PMC6522624; doi:10.1186/s41181-019-0060-9)
Supplement: Supplementary file 1 — Figure S1. Schematic representation of the peptides investigated in the study. Figure S2. RP-HPLC chromatograph of [68Ga]Ga-Ex4NOD (top) and [68Ga]Ga-Ex4DFO (bottom). Figure S3. Mass spectrometric analysis of [natGa]Ga-Ex4NOD. Figure S4. Mass spectrometric analysis of [natGa]Ga-Ex4DFO. (DOCX 812 kb) [file 41181_2019_60_MOESM1_ESM.docx]

Supplementary information to

**Comparison of desferrioxamine and NODAGA for the gallium-68 labeling of exendin-4**

Simon A.M. Kaeppeli^1^, Roger Schibli^1,2^, Thomas L. Mindt^3,4^, Martin Behe^1^

^1^Center for Radiopharmaceutical Sciences ETH-PSI-USZ, Paul Scherrer Institute, Forschungsstrasse 111, 5232 Villigen-PSI, Switzerland.

^2^Department of Chemistry and Applied Biosciences, ETH Zurich, Vladimir-Prelog-Weg 4, 8093 Zurich, Switzerland.

^3^Ludwig Boltzmann Institute Applied Diagnostics, General Hospital Vienna (AKH), c/o Sekretariat Nuklearmedizin Währinger Gürtel 18-20, Vienna, Austria.

^4^Department of Biomedical Imaging and Image Guided Therapy, Division of Nuclear Medicine, Medical University of Vienna, Vienna, Austria

**Corresponding author:**

Dr. Martin Behe, OIPA/102, Forschungsstrasse 111, 5232 Villigen-PSI, Switzerland

[martin.behe@psi.ch](mailto:martin.behe@psi.ch), Tel +41 56 310 2817, Fax +41 56 310 2849


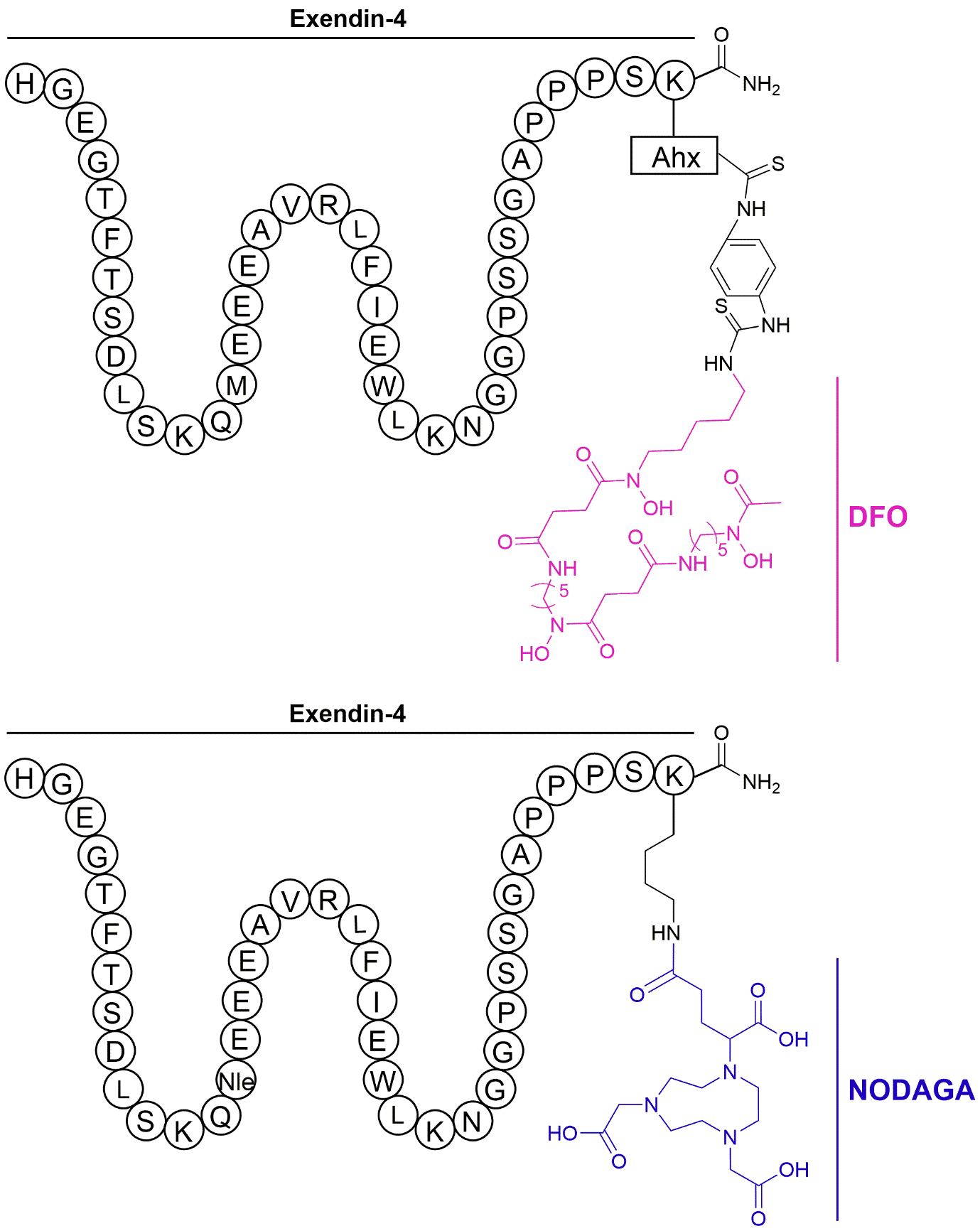


**Figure S1. Schematic representation of the peptides investigated in the study.** The top shows Ex4DFO and the bottom shows Ex4NOD. The chelators are depicted without coordinated radiometal.


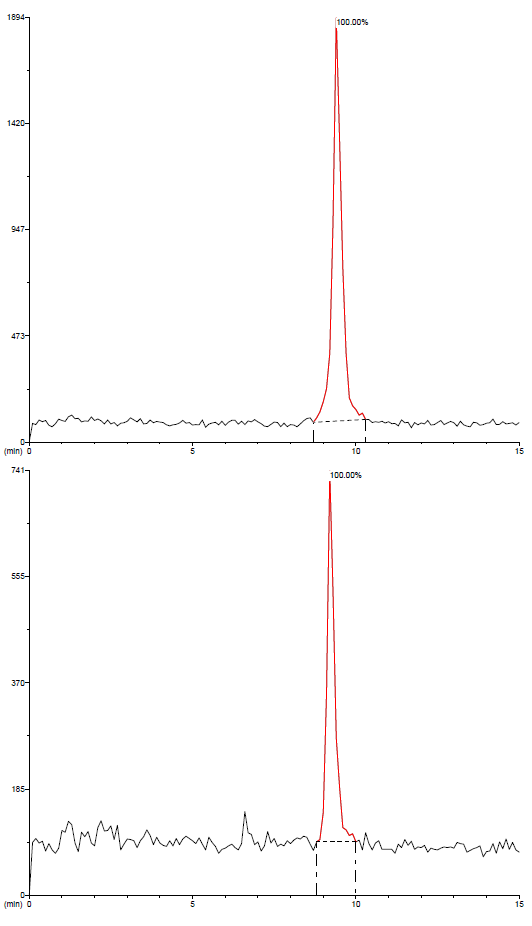


**Figure S2. RP-HPLC chromatograph of [^68^Ga]Ga-Ex4NOD (top) and [^68^Ga]Ga-Ex4DFO (bottom).** The NODAGA-conjugated peptide eluted at 9.40 min and the DFO-conjugated derivative eluted at 9.20 min. No free gallium-68 could be observed.


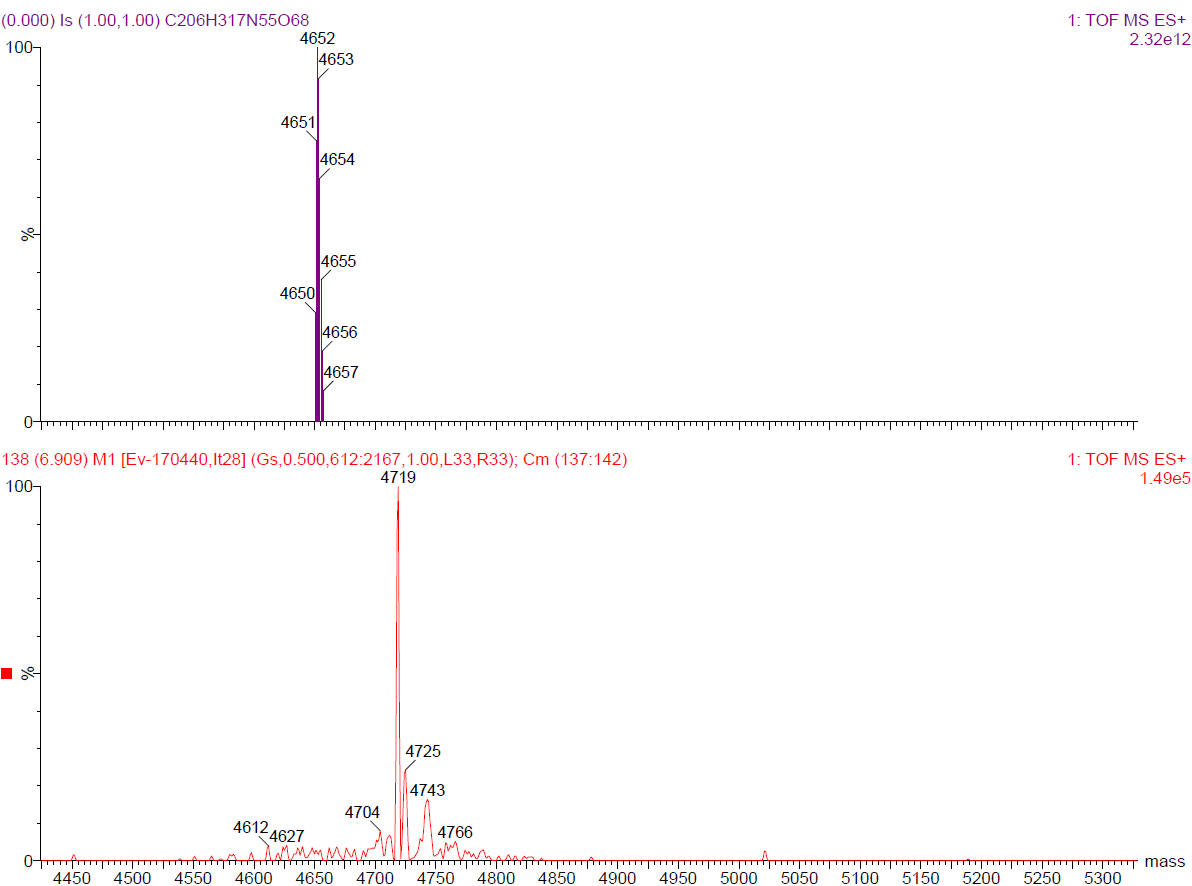


**Figure S3. Mass spectrometric analysis of [^nat^Ga]Ga-Ex4NOD.** Top: Theoretical mass determination of unlabeled peptide, bottom: Mass distribution of labeled peptide.


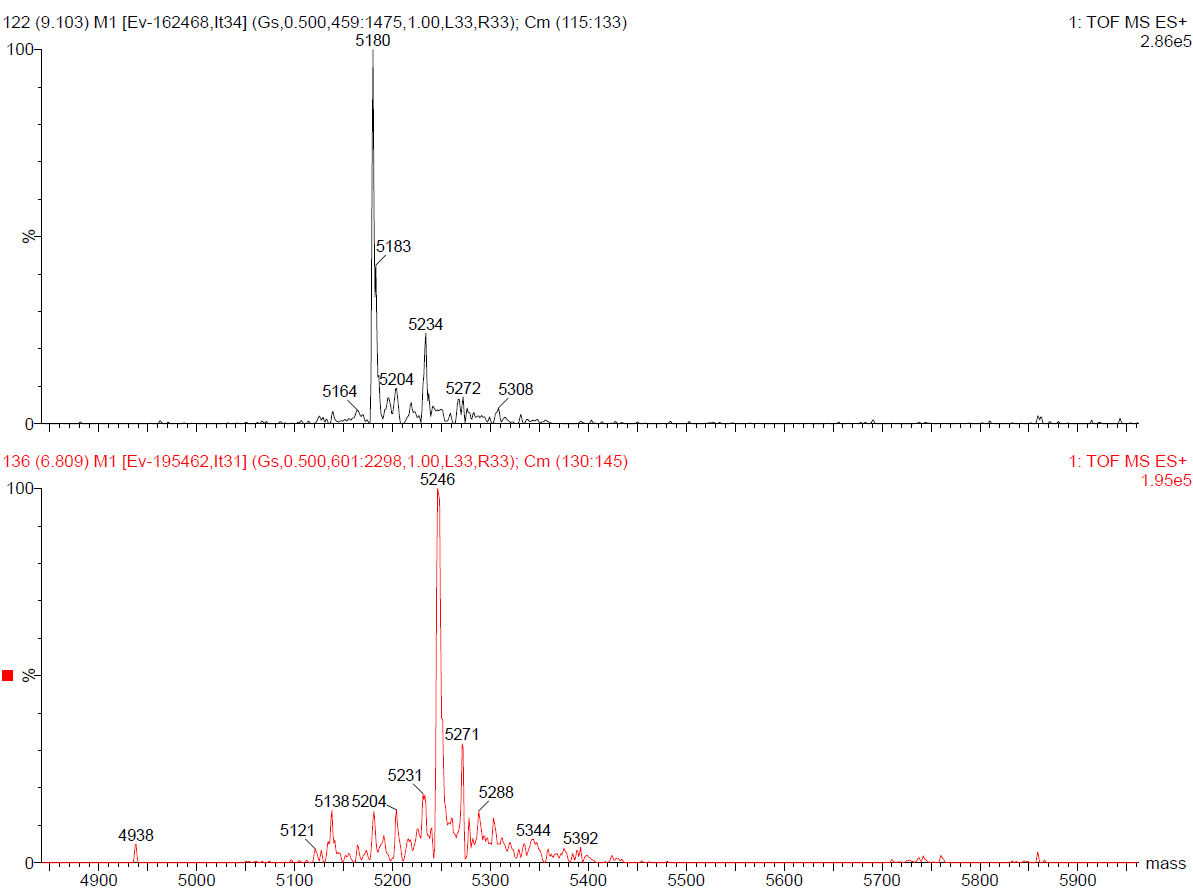


**Figure S4. Mass spectrometric analysis of [^nat^Ga]Ga-Ex4DFO.** Top: Mass distribution of unlabeled peptide, bottom: Mass distribution of labeled peptide.
